# Unrelated Donor Cord Blood Transplantation in Children: Lessons Learned Over 3 Decades

**DOI:** 10.1093/stcltm/szac079

**Published:** 2023-01-30

**Authors:** Joanne Kurtzberg, Jesse D Troy, Kristin M Page, Hanadi Rafii El Ayoubi, Fernanda Volt, Graziana Maria Scigliuolo, Barbara Cappelli, Vanderson Rocha, Annalisa Ruggeri, Eliane Gluckman

**Affiliations:** The Marcus Center for Cellular Cures, Duke University School of Medicine, Durham, NC, USA; The Marcus Center for Cellular Cures, Duke University School of Medicine, Durham, NC, USA; Division of Pediatric Hematology/Oncology/BMT at the Medical College of Wisconsin, Milwaukee, WI, USA; Eurocord, Hopital Saint Louis APHP, Institut de Recherche de Saint-Louis (IRSL) EA3518, Université de Paris Cité, Paris, France; Monacord, Centre Scientifique de Monaco, Monaco; Eurocord, Hopital Saint Louis APHP, Institut de Recherche de Saint-Louis (IRSL) EA3518, Université de Paris Cité, Paris, France; Eurocord, Hopital Saint Louis APHP, Institut de Recherche de Saint-Louis (IRSL) EA3518, Université de Paris Cité, Paris, France; Monacord, Centre Scientifique de Monaco, Monaco; Eurocord, Hopital Saint Louis APHP, Institut de Recherche de Saint-Louis (IRSL) EA3518, Université de Paris Cité, Paris, France; Monacord, Centre Scientifique de Monaco, Monaco; Eurocord, Hopital Saint Louis APHP, Institut de Recherche de Saint-Louis (IRSL) EA3518, Université de Paris Cité, Paris, France; Service of Hematology, Transfusion and Cell Therapy, and Laboratory of Medical Investigation in Pathogenesis and Directed Therapy in Onco-Immuno-Hematology (LIM-31), Hospital das Clínicas, Faculty of Medicine, São Paulo University (FM-USP), São Paulo, Brazil; Eurocord, Hopital Saint Louis APHP, Institut de Recherche de Saint-Louis (IRSL) EA3518, Université de Paris Cité, Paris, France; Haematology and Bone Marrow Transplant Unit, IRCCS San Raffaele Scientific Institute, Milan, Italy; Eurocord, Hopital Saint Louis APHP, Institut de Recherche de Saint-Louis (IRSL) EA3518, Université de Paris Cité, Paris, France; Monacord, Centre Scientifique de Monaco, Monaco

## Abstract

Four decades ago, Broxmeyer et al. demonstrated that umbilical cord blood (CB) contained hematopoietic stem cells (HSC) and hypothesized that CB could be used as a source of donor HSC for rescue of myeloablated bone marrow. In 1988, Gluckman et al. reported the first successful matched sibling cord blood transplant (CBT) in a child with Fanconi Anemia. In 1991, Rubinstein et al. established an unrelated donor CB bank, and in 1993, the first unrelated CBT used a unit from this bank. Since that time, >40 000 CBTs have been performed worldwide. Early outcomes of CBT were mixed and demonstrated the importance of cell dose from the CB donor. We hypothesized that improvements in CB banking and transplantation favorably impacted outcomes of CBT today and performed a retrospective study combining data from Eurocord and Duke University in 4834 children transplanted with a single unrelated CB unit (CBU) from 1993 to 2019. Changes in standard transplant outcomes (overall survival [OS], disease free survival [DFS], acute and chronic graft-versus-host disease [GvHD], treatment related mortality [TRM], and relapse) over 3 time periods (1: <2005; 2: 2005 to <2010; and 3: >2010 to 2019) were studied. Increased cell dose and degree of HLA matching were observed over time. OS, times to engraftment, and DFS improved over time. The incidence of TRM and GvHD decreased while the incidence of relapse remained unchanged. Relative contributions of cell dose and HLA matching to transplant outcomes were also assessed and showed that HLA matching was more important than cell dose in this pediatric cohort.

## Graphical Abstract

**Graphical Abstract:**
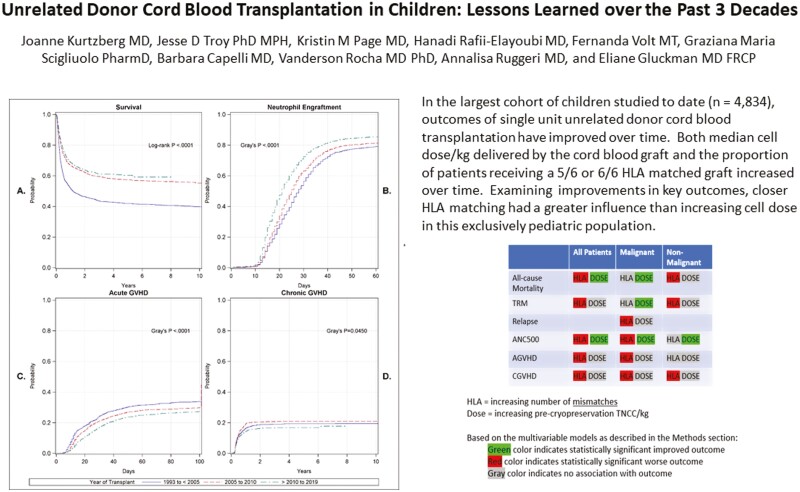

**Significance Statement**

This article presents the largest report of outcomes of unrelated donor cord blood transplantation in pediatric patients with malignant and non-malignant conditions over nearly 30 years, starting with the first unrelated donor cord blood transplant ever performed in the world. Analysis of this large dataset identified changes in practice, over time, that have improved outcomes and answered critical questions about HLA, cell dose, and the use of TBI and ATG in conditioning regimens, which have not been answerable in past studies using smaller patient datasets.

## Introduction

Forty years ago, Hal Broxmeyer discovered that umbilical cord blood (CB) contained hematopoietic stem and progenitor cells (HSCs),^1^ leading to the use of CB as an alternative donor for hematopoietic stem cell transplantation (HSCT). CB is rapidly available, enables permissive HLA-matching, low rates of graft versus host disease (GvHD), and enhanced graft versus leukemia (GvL) effects. Historically, CB was associated with slower hematopoietic engraftment and immune reconstitution, higher infectious complications and transplant-related mortality (TRM).^2-4^ Early trials demonstrated that cell dose (CD) was rate limiting for engraftment,^2,3^ The fact that CB is the only banked source of HSCs led to regulation as a commercial product resulting in increased costs compared to nonregulated HSC sources.

Outcomes of CBT have improved since the early days of implementation.^2-8^ Publicly banked CBUs are larger^9^ resulting in shorter times to engraftment and reduced TRM. The use of dCBT and the emergence of ex-vivo expansion technology have decreased time to engraftment, days of hospitalization, and TRM.^10^ In experienced centers, outcomes of CBT are equivalent or superior to outcomes of HSCT using other donor sources.^7,11-13^

Despite these benefits, the use of CBT has decreased worldwide. Much of this decline is related to the use of haplo-identical related donors enable by the development of innovative strategies to prevent GvHD.^14^ In light of these trends, we investigated outcomes after CBT in a large international cohort of children treated over the past 3 decades. We asked key questions about the interactions between HLA matching and cell dose (CD), the role of anti-thymocyte globulin (ATG), and the role of total body irradiation (TBI) in patients with leukemia.

## Methods

This multicenter retrospective study was conducted through a joint collaboration with Eurocord/European Blood and Marrow Transplant Group (EBMT) and the Pediatric Transplant and Cellular Therapy Program at Duke University. The aim of the study was to describe trends in outcomes of unrelated CBT in children over 28 years.

### Study Design

Eligible patients were children (<18 years of age) who received an unrelated single unit CBT, as their first allogeneic HSCT between 1993 and 2019, for hematological malignancies and non-malignant diseases. Patients who received other products or a second allogeneic transplant were excluded. Data related to patient demographics (excluding race/ethnicity, which is not permitted to be collected in France), CBU characteristics, and transplant outcomes were analyzed. The Institutional Review Boards of the Eurocord scientific committee and Duke University approved this study. All patients or their parents or legal guardians gave informed consent for treatment, data entry, and analysis in accordance with the Declaration of Helsinki.

### Endpoints and Definitions

The primary endpoint was overall survival (OS), defined as the probability of being alive regardless of disease status. Secondary outcomes included the cumulative incidence (CI) of neutrophil recovery, acute and chronic GvHD (aGvHD and cGvHD), GvHD, Relapse Free, Survival (GFRF), and TRM, defined as any death not caused by relapse or persistent malignancy. Disease-free survival (DFS), defined as survival while in continuous complete remission (CR), and relapse were evaluated in patients with malignant diseases. Surviving patients who did not experience any of these events were censored at the time of last follow-up. Neutrophil engraftment was defined as the achievement of a sustained absolute neutrophil count greater than 0.5 × 10^9^/L for 3 consecutive days. Graft failure was defined as failure to achieve neutrophil engraftment, loss of donor engraftment, or autologous recovery within day +100 after CBT. The diagnosis and grading of acute and chronic GvHD were assigned by the transplant center using standard criteria. The conditioning regimen was defined as myeloablative (MAC) or reduced intensity (RIC) based on EBMT and CIBMTR criteria. CD was calculated as the total number of pre-cryopreservation and infused total nucleated cells (TNC)/kg and CD34 cells/kg, respectively. Human leukocyte antigen (HLA) matching between the recipient and the UCB donor was determined conventionally considering antigen-level HLA typing (low resolution) for Class I-A and -B loci and allele-level (high resolution) for Class II-DRB1 locus. A subset of patients had HLA allelic typing.

### Statistical Analysis

Calendar time was divided into 3 periods for analysis of transplant outcomes: P1—1990 to <2005, P2—2005 to <2010, and P3—2010 to 2019. Overall survival (OS) was analyzed using the Kaplan–Meier method. TRM, relapse, acute, and chronic GvHD, and time to neutrophil recovery were analyzed using the CI method for competing risks. Competing events were relapsed (in the analysis of TRM) or death (in the analysis of relapse, neutrophil recovery, and acute and chronic GvHD). Unadjusted comparisons of these outcomes across calendar periods were accomplished using the log-rank test (OS) or Gray’s test (all other outcomes). Multivariable models were fit describing the association between calendar period and each of the outcomes after adjustment for use of ATG, HLA mismatch, age at transplant, pre-transplant serum positivity for cytomegalovirus (CMV), malignant versus non-malignant indication for transplant, pre-cryopreservation TNC dose/kg of patient weight (TNC/kg), MAC vs. RIC conditioning regimen, and use of TBI. All covariates were included in every model regardless of statistical significance to allow evaluation of the association between each covariate and the transplant outcomes after adjustment for the other factors. Cox proportional hazards regression was used to model overall survival and the Fine and Gray model was used for the other outcomes that have competing events. A total of 33.9% of participants were missing data on at least one of the model covariates, with the highest missingness rate for an individual covariate being 16.7%. Missingness in covariates was related to calendar period (9.6%, 29.5%, and 55.8% missing at least one covariate in the early (P1), middle (P2), and later (P3) periods, respectively) and was modestly associated with older age at transplant and malignant disease. Prior to fitting any models, all missing covariate data were imputed (20 datasets)^15^ using the fully conditional specification method. Imputations of continuous variables were restricted to be within the range of the observed data. Analysis of OS (no missing outcomes), neutrophil engraftment, and relapse (missing in <1% of participants) were based on models fit to the available data after covariate imputation. Analysis of TRM, aGvHD, and cGvHD was based on inverse probability weighted models (IPW)^16^ as these outcomes were missing in 10–25% of participants. Weights were estimated (after covariate imputation) using logistic regression as the inverse of the predicted probability of having an observed outcome based on the covariates used in the analysis model described above. Results of models fit to each of the imputed datasets were combined using Rubin’s rules^17^ and summary hazard ratios (HR) and 95% CIs were reported. All analyses were conducted using SAS v9.4 (Cary, NC) with PROC MI, MIANALYZE, PHREG, and GENMOD.

## Results

### Patient, Disease, and Transplant Characteristics

A total of 4834 patients were analyzed, 4015 from the Eurocord database and 819 from Duke (Table 1). The number of patients in the respective time periods were: P1:1993–<2005 (*n* = 1297), P2:2005–2010 (*n* = 1735), P3:>2010–2019 (*n* = 1802). Most patients, 58.7%, were transplanted for a malignant disease (*n* = 2839) of whom 2347 had acute leukemia [acute lymphoblastic leukemia (ALL), 1422; acute myelogenous leukemia (AML), 887], and 167 myelodysplasia (MDS). The remaining 1995 patients (41.3%) were transplanted for a non-malignant disease: 761 (15.7%) for Inborn Errors of Metabolism (IEM); 644 (13.3%) for primary immunodeficiency disease (ID); and 325 (6.7%) for bone marrow failure syndromes. Pre-transplant recipient CMV serology was positive in 49.4%. There was a decrease in the number of patients transplanted for malignant diagnoses and an increase in patients transplanted for non-malignant diagnoses over time. The majority (84.6%) were prepared for transplant with a MAC regimen. A reduction in the use of TBI in patients with leukemia was observed, from 42.5% in P1 to 19.7% in P3.

**Table 1.** Patient characteristics by time period.

	1993 to <2005 (*N* = 1297)	2005-2010 (*N* = 1735)	>2010-2019 (*N* = 1802)	Total (*N* = 4834)
Age at transplant				
Mean (SD)	6.24 (4.95)	5.07 (4.58)	4.85 (4.49)	5.30 (4.68)
Median	5.20	3.50	3.25	3.80
Range	(0.10-17.90)	(0.10-17.90)	(0.10-17.90)	(0.10-17.90)
Sex				
Male	778 (60.0%)	1031 (59.7%)	1032 (58.0%)	2841 (59.2%)
Female	519 (40.0%)	695 (40.3%)	746 (42.0%)	1960 (40.8%)
Missing	0 (0.0%)	9 (<0.1%)	24 (<0.1%)	33 (<0.1%)
Disease type				
Non-malignant	417 (32.2%)	738 (42.5%)	840 (46.6%)	1995 (41.3%)
Malignant	880 (67.8%)	997 (57.5%)	962 (53.4%)	2839 (58.7%)
Indication for transplant				
Acute leukemia	725 (55.9%)	816 (47.0%)	806 (44.7%)	2347 (48.6%)
MDS	53 (4.1%)	55 (3.2%)	59 (3.3%)	167 (3.5%)
MPD	32 (2.5%)	22 (1.3%)	18 (1.0%)	72 (1.5%)
MDS/MPD	34 (2.6%)	61 (3.5%)	47 (2.6%)	142 (2.9%)
Lymphoproliferative disorder	29 (2.2%)	37 (2.1%)	28 (1.6%)	94 (1.9%)
Histiocytic disorder	33 (2.5%)	94 (5.4%)	79 (4.4%)	206 (4.3%)
Solid tumor	7 (0.5%)	6 (0.3%)	4 (0.2%)	17 (0.4%)
Bone marrow failure syndrome	89 (6.9%)	118 (6.8%)	118 (6.5%)	325 (6.7%)
Hemoglobinopathy	8 (0.6%)	17 (1.0%)	20 (1.1%)	45 (0.9%)
Primary immune deficiency	98 (7.6%)	239 (13.8%)	307 (17.0%)	644 (13.3%)
Inborn error of metabolism	188 (14.5%)	267 (15.4%)	306 (17.0%)	761 (15.7%)
Autoimmune disease	1 (0.1%)	3 (0.2%)	10 (0.6%)	14 (0.3%)
Recipient CMV status at transplant				
Negative	681 (55.3%)	782 (49.4%)	751 (45.4%)	2214 (49.5%)
Positive	541 (43.9%)	783 (49.4%)	886 (53.6%)	2210 (49.4%)
Not performed	10 (0.8%)	19 (1.2%)	17 (1.0%)	46 (1.0%)
Missing	65 (<0.1%)	151 (<0.1%)	148 (<0.1%)	364 (<0.1%)
Conditioning regimen				
MAC	1162 (91.4%)	1338 (79.7%)	1427 (84.3%)	3927 (84.6%)
Cyclophosphamide	952 (73.4%)	1204 (69.4%)	820 (45.5%)	2976 (61.6%)
Fludarabine	110 (8.5%)	602 (34.7%)	1015 (56.3%)	1727 (35.7%)
RIC	110 (8.6%)	340 (20.3%)	265 (15.7%)	715 (15.4%)
Missing	25 (<0.1%)	57 (<0.1%)	110 (<0.1%)	192 (<0.1%)
Total body irradiation				
No	743 (57.5%)	1254 (72.9%)	1372 (80.3%)	3369 (71.4%)
Yes	549 (42.5%)	466 (27.1%)	337 (19.7%)	1352 (28.6%)
Missing	5 (<0.1%)	15 (<0.1%)	93 (<0.1%)	113 (<0.1%)
GvHD prophylaxis				
CSA	415 (33.2%)	300 (18.6%)	241 (14.6%)	956 (21.2%)
CsA + MMF ± steroids/pred	14 (1.1%)	438 (27.2%)	630 (38.2%)	1082 (24.0%)
CsA + MTX ± Other	88 (7.0%)	82 (5.1%)	78 (4.7%)	248 (5.5%)
CsA + pred	665 (53.2%)	692 (43.0%)	608 (36.9%)	1965 (43.6%)
FK506 ± other	30 (2.4%)	73 (4.5%)	65 (3.9%)	168 (3.7%)
Other	38 (3.0%)	26 (1.6%)	26 (1.6%)	90 (2.0%)
Missing	47 (<0.1%)	124 (<0.1%)	154 (<0.1%)	325 (<0.1%)
ATG				
No	96 (7.6%)	322 (20.0%)	160 (11.9%)	578 (13.7%)
Yes	1171 (92.4%)	1284 (80.0%)	1179 (88.1%)	3634 (86.3%)
Missing	30 (<0.1%)	129 (<0.1%)	463 (<0.1%)	622 (<0.1%)

The TNC dose/kg increased significantly over time with a median precryopreservation dose/kg from 6.66 × 10^7^ in P1 to 9.29 × 10^7^ in P3 (*P* = <.0001) (Table 2). The same trends were observed with the infused TNC and CD34 cell doses. We were not able to include infused TNC or CD34 cell doses in the multivariate analyses because of missing data (for infused TNC) and collinearity concerns for CD34. Both the precryopreservation and infused TNC/kg (median 8.07 and 6.17 × 10^7^) and infused CD34 cell doses/kg (median 2.30 × 10^5^) were high in this pediatric cohort.

**Table 2.** Cord blood characteristics.

	1993 to < 2005 (*N* = 1297)	2005 to 2010 (*N *= 1735)	>2010 to 2019 (*N* = 1802)	Total (*N* = 4834
Pre-cryo TNCC/kg, ×10e7				
Missing	31	263	514	808
Mean (SD)	8.48 (6.79)	11.02 (8.66)	11.89 (8.65)	10.50 (8.23)
Median	6.66	8.38	9.29	8.07
Range	(0.67-65.06)	(0.38-101.25)	(0.45-81.36)	(0.38-101.25)
Infused TNCC/kg, ×10e7				
*N*	31	263	514	808
Mean (SD)	6.35 (4.96)	8.28 (6.24)	9.06 (6.68)	7.92 (6.12)
Median	5.00	6.52	7.13	6.17
Range	(0.59-48.50)	(0.30-81.00)	(0.33-65.09)	(0.30-81.00)
Infused CD34/kg, ×10e5				
Missing	129	353	589	1,071
Mean (SD)	3.69 (5.91)	3.25 (3.35)	3.71 (3.68)	3.53 (4.40)
Median	2.00	2.30	2.50	2.30
Range	(0.05-91.53)	(0.04-45.00)	(0.05-27.89)	(0.04-91.53)
Low resolution HLA match				
Missing	73	259	339	671
6/6	123 (10.0%)	322 (21.8%)	353 (24.1%)	798 (19.2%)
5/6	514 (42.0%)	740 (50.1%)	779 (53.2%)	2033 (48.8%)
4/6	526 (43.0%)	395 (26.8%)	308 (21.1%)	1229 (29.5%)
3/6	55 (4.5%)	18 (1.2%)	22 (1.5%)	95 (2.3%)
2/6	6 (0.5%)	1 (0.1%)	1 (0.1%)	8 (0.2%)
High resolution HLA match				
Missing	1173	1316	879	3368
8/8	8 (6.5%)	34 (8.1%)	130 (14.1%)	172 (11.7%)
7/8	15 (12.1%)	80 (19.1%)	192 (20.8%)	287 (19.6%)
6/8	29 (23.4%)	93 (22.2%)	279 (30.2%)	401 (27.4%)
5/8	31 (25.0%)	111 (26.5%)	200 (21.7%)	342 (23.3%)
4/8	22 (17.7%)	64 (15.3%)	92 (10.0%)	178 (12.1%)
3/8	16 (12.9%)	30 (7.2%)	23 (2.5%)	69 (4.7%)
2/8	3 (2.4%)	7 (1.7%)	7 (0.8%)	17 (1.2%)

HLA matching was scored at low resolution for Class I HLA-A, -B and high resolution for Class II (HLA-DRB1) for all patients. As technology for HLA typing advanced over time, high resolution Class I HLA-A, -B, and -C and Class II HLA-DRB1 typing were performed and considered. Whether low- or high-resolution typing was evaluated, the use of more closely matched CBUs increased over time. The numbers of patients receiving 5-6 of 6 HLA matched grafts increased from 52.1% before 2005 to 74.9% after 2010 (Supplementary Fig. S4). There was also an increase in DRB1 matching from P1 to P3 76.4% increasing to 82.7%. HLA by high resolution typing was available for 1466 patient/CB pairs at 8 loci and the same trend to more HLA compatibility was observed with a doubling of patients receiving grafts with 0 or 1 HLA mismatches (*P* = <.0001) (Table 2, Supplementary Fig. S1).

### Overall Survival by Year of Transplant

**Figure 1.**
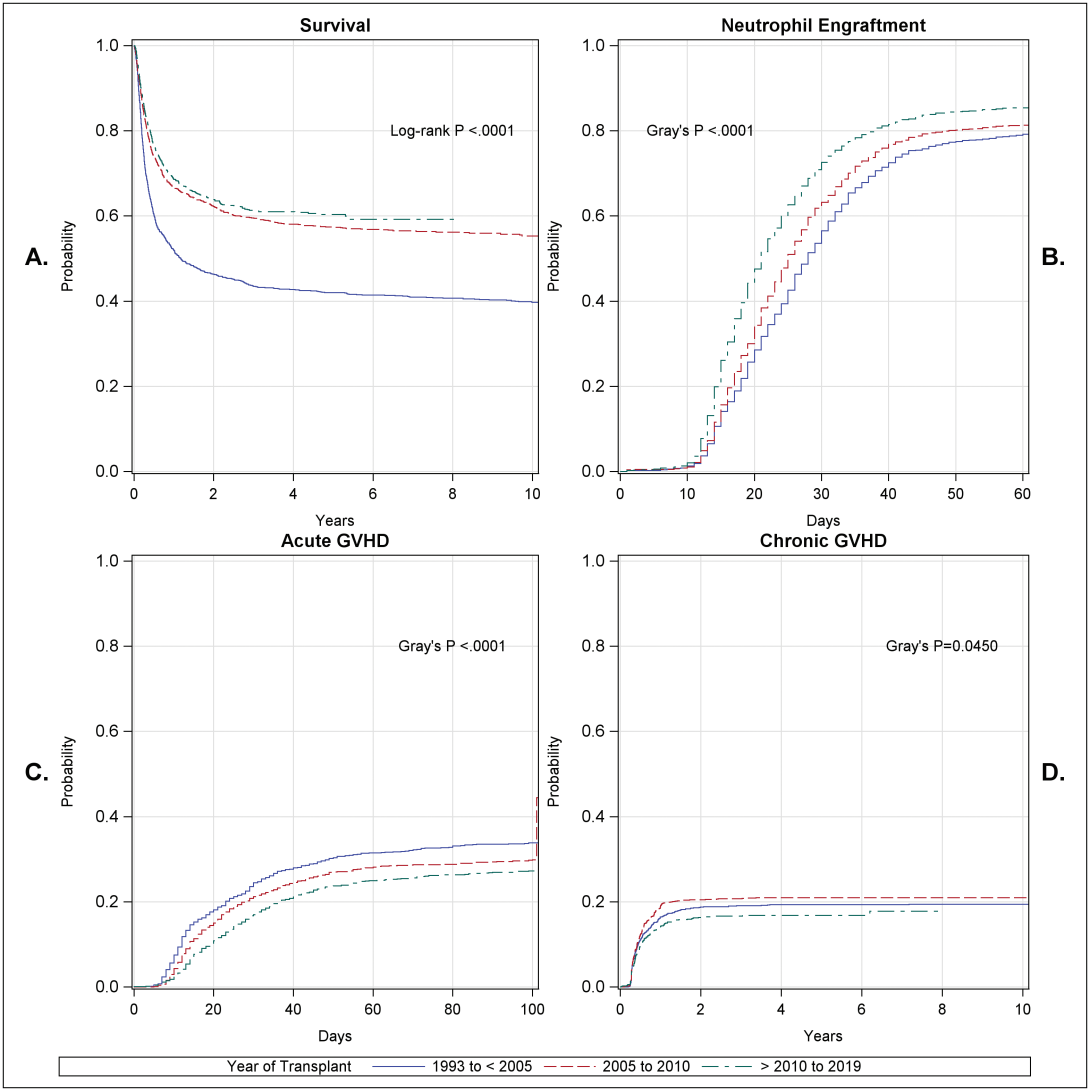
Transplant outcomes by time period: overall survival (**A**), neutrophil engraftment (**B**), acute GvHD (**C**), and chronic GvHD (**D**) over time for period 1 (1993 to <2005, blue), period 2 (2005-2010, red), and period 3 (>2010-2019, green). Overall survival increased between period 1 and periods 2 and 3. Neutrophil engraftment increased incrementally over all 3 periods. Acute GvHD decreased incrementally from the earliest to the later time periods. Chronic GvHD was relatively stable over time.

**Figure 2.**
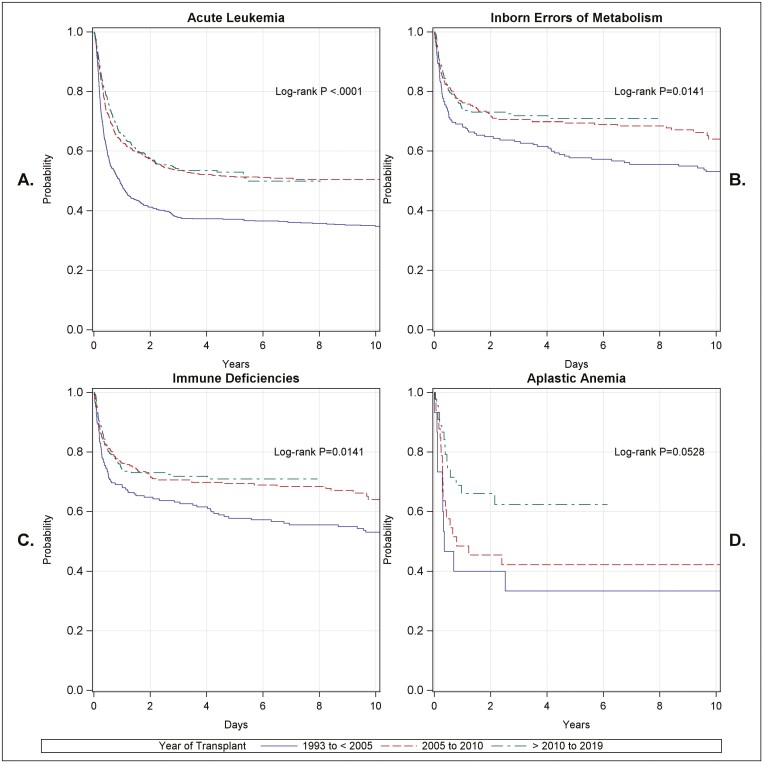
Survival by time in selected disease groups: Overall survival over time in patient cohorts with acute leukemia (**A**), inborn errors of metabolism (**B**), immunodeficiency syndromes (**C**), and severe aplastic anemia (**D**). Survival increased from the earliest to the latest time periods in all diseases, with outcomes in acute leukemia having the largest increase over time. Improvements in survival in patients with aplastic anemia were predominantly seen in the most recent time period.

The probability of 5-year OS in the entire cohort was 53.8% (95% CI: 52.2%, 55.3%) (Supplementary Fig. S2A). OS improved significantly over time: 42% in P1 to 60.4% in P3 (*P* = <.0001) (Fig. 1A). OS by major diagnostic categories (Fig. 2, Supplementary Fig. S3) had the largest increases in patients with leukemia followed by IEM and ID. The probability of OS for patients with leukemia at 5 years was 47.2% (95% CI: 45.0%, 49.3%) (Supplementary Fig. S3A) and increased from 37.1% (95% CI: 33.6, 40.6) in P1 to 52.9% (95% CI: 48.5, 57.1) in P3 (*P* < .0001) (Fig. 2A). The probability of OS for the 761 patients with IEM was 66.5% (95% CI: 62.8%, 69.9%) (Supplementary Fig. S3B) and increased over time from 57.8% (95% CI: 50.4, 64.5) in P1 to 71% (95% CI: 64.9, 76.2) in P3 (*P* = .01) (Fig. 2B). The probability of OS of the 644 patients with ID was 63.1% (95% CI: 58.8%, 67%) (Supplementary Fig. S3C) and increased over time from 55.4% (95% CI: 44.9, 64.7) to 62.1% (95% CI: 54.8, 68.6) (Fig. 2C). The probability of OS for the 93 patients with severe aplastic anemia was 54.1% (95% CI: 43.1%, 63.8%) and increased from 33.3% in P1 (95% CI: 23.3, 56.4) to 62.4% (95% CI: 45.2, 75.7) in P3 (0.0528) (Fig. 2D, Supplementary Fig. S3D).

### Causes of Death

**Figure 3.**
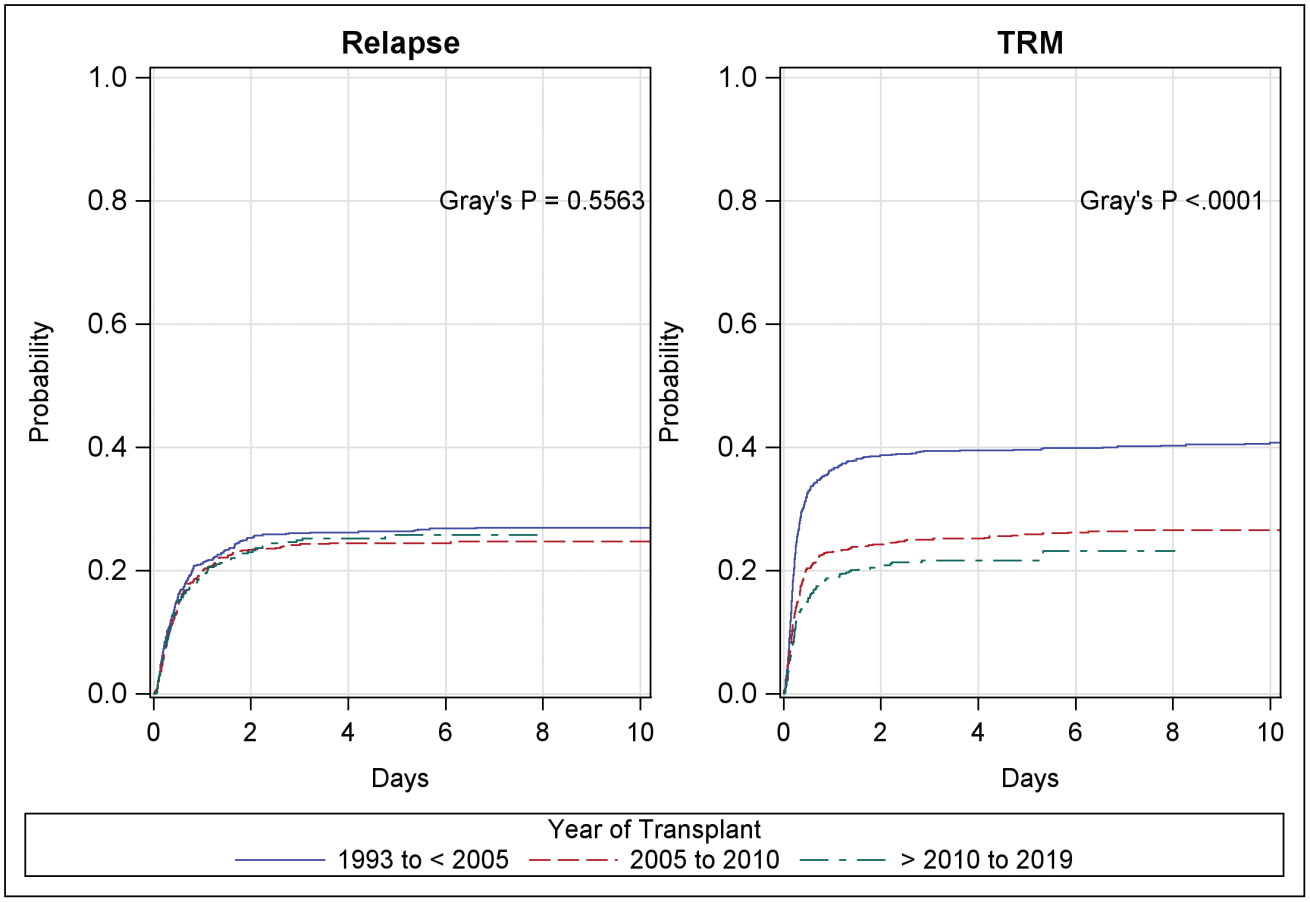
Relapse and TRM in patients with malignant diagnoses: The incidence of relapse remained constant over the 3 time periods examined. The incidence of TRM decreased over time.

The causes of death by year of transplant are shown in Supplementary Table S1. The main causes of death were relapse/disease progression (*n* = 628, 30.1%) and TRM including GvHD (*n* = 1220, 58.4%), secondary malignancy (*n* = 12), other or missing (*n* = 229). GvHD was responsible for TRM in 19.6% of patients overall and was relatively stable over the 3 time periods (18.2%, 18.7%, and 22.6%) studied (Supplementary Table S2). In patients with malignancies, rates of death from relapse remained stable while TRM decreased over time (Fig. 3), but TRM remained the leading cause of death in patients with malignancies over time. Earlier year of transplantation (*P* = <.001), increasing HLA mismatch (*P* = <.002), positive CMV serology pretransplant (*P* = .001), precryo TNCC (*P* = .18), and older age at transplant (*P* = .011) were associated with increased risk of death (Table 3). There was also a trend (*P* = .66) toward increased mortality with use of ATG (Supplementary Table S3).

**Table 3.** Results of Cox proportional hazards regression modeling of death from any cause.

	All transplants		Malignant		Non-malignant	
	(*N* = 4834)		(*N* = 2839)		(*N *= 1995)	
	HR (95% CI)	*P*-value	HR (95% CI)	*P*-value	HR (95% CI)	*P*-value
Year of transplant		<.001		<.001		<.001
1993 to < 2005	Ref		Ref		Ref	
2005 to 2010	0.66 (0.59,0.73)		0.61 (0.54,0.70)		0.73 (0.60,0.89)	
> 2010 to 2019	0.61 (0.54,0.68)		0.54 (0.47,0.63)		0.69 (0.57,0.85)	
ATG		.677		.245		.106
No	Ref		Ref		Ref	
Yes	1.03 (0.90,1.18)		0.92 (0.78,1.08)		1.23 (0.95,1.59)	
HLA mismatch		<.001		.203		<.001
5/6, 6/6	Ref		Ref		Ref	
≥ 4/6	1.18 (1.07,1.30)		1.08 (0.96,1.21)		1.37 (1.15,1.64)	
Age, years		.074		.226		.008
<5	Ref		Ref		Ref	
≥ 5 to < 12	1.02 (0.91,1.14)		1.01 (0.88,1.15)		1.05 (0.85,1.29)	
≥ 12	1.19 (1.01,1.40)		1.14 (0.96,1.37)		1.77 (1.22,2.58)	
CMV		<.001		<.001		<.001
Negative	Ref		Ref		Ref	
Positive	1.40 (1.28,1.54)		1.42 (1.27,1.59)		1.36 (1.17,1.60)	
Diagnosis		<.001	—	—	—	—
Non-malignant	Ref		—	—	—	—
Malignant	1.35 (1.21,1.50)		—	—	—	—
Pre-cryo TNCC, × 10e7/kg		.028		.014		.759
<2.5	Ref		Ref		Ref	
2.5-5	0.78 (0.62,0.98)		0.72 (0.56,0.93)		1.08 (0.56,2.06)	
>5	0.74 (0.58,0.94)		0.68 (0.52,0.89)		1.01 (0.53,1.92)	
Conditioning		.011		.065		.065
MAC	Ref		Ref		Ref	
RIC	1.18 (1.03,1.35)		1.21 (0.98,1.50)		1.17 (0.98,1.40)	
TBI		.362		.086	—	—
No	Ref		Ref		—	—
Yes	0.95 (0.85,1.06)		0.90 (0.80,1.01)		—	—

All models are based on multiply imputed covariate data (20 datasets). See the Methods section for complete details.

Abbreviations: HR, hazard ratio; CI, confidence interval.

### Engraftment by Year of Transplant

Times to engraftment of neutrophils and platelets shortened over time (Fig. 1B, Supplementary Fig. S2B). The median times to neutrophil and platelet engraftment in the entire cohort were 22 days (25th-75th percentile 16-29) and 43 days (25th-75th percentile 33-61), respectively. The median time to neutrophil engraftment was 25 days in P1 and 19 days in P3. The median day of platelet engraftment was 54.5 days in P1 and 38 days in P3. The 60-day CI probability of neutrophil engraftment in the entire cohort was 83.3% (85% CI: 82.2%, 84.3%) and improved over time; 76.9% in P1 to 85.3% in P3 (*P* = <.0001). In multivariable analysis, calendar period (HR = 1.09, 95% CI: 1.01, 1.18 and HR = 1.37, 95% CI: 1.26, 1.49 comparing P2 and P3 vs. P1 respectively; *P* < .001), higher cell dose (HR=1.11, 95% CI: 0.91, 1.36, and HR = 1.39, 95% CI: 1.13, 1.71 for 2.5-5 and >5 vs. <2.5 × 10e^7^/kg; *P* < .001), and diagnosis with malignant vs. non-malignant disease (HR = 1.08, 95% CI: 1.00, 1.16; *P* = .054) were factors favorably associated with neutrophil engraftment. HLA mismatch (HR = 0.9, 95% CI: 0.83, 0.97 comparing ≤4/6 vs. 5/6 and 6/6), older age (HR = 0.91, 95% CI: 0.84, 0.98, and HR = 0.99, 95% CI: 0.88, 1.11 for 5-12 and ≥12 vs. <5 years old; *P* = .03), positive vs. negative CMV serology (HR = 0.85, 95% CI: 0.8, 0.91; *P* < .001), and RIC vs. MAC conditioning (HR = 0.87, 95% CI: 0.79, 0.96; *P* = .006) were negatively associated with neutrophil engraftment. ATG use (HR = 1.02, 95% CI: 0.92, 1.12; *P* = .699) and TBI (HR = 1.05, 95% CI: 0.97, 1.14; *P* = .217) were unrelated to neutrophil engraftment in the multivariable model.

### Acute and Chronic GvHD

The incidence of acute (at day 100) and chronic (at 1 year) GvHD in the entire cohort was 30.1% (95% CI: 28.7, 31.4) and 16.7% (95% CI: 15.4, 17.9) respectively (Fig. 1C and 1D, Supplementary Fig. S2C and S2D). Both aGvHD and cGvHD decreased over time (*P* = <.001 for acute and *P* = .045 for chronic). In multivariable analysis, decreased aGvHD was associated with better HLA matching and with the use of ATG (Table 4) and decreased cGvHD showed a positive association with younger age, better HLA match, negative CMV serology and diagnosis of a malignant disease (Table 4).

**Table 4.** Results of fine and gray proportional hazards regression modeling of acute and chronic graft versus host disease (GvHD).

	Acute GvHD		Chronic GvHD	
	(*N* = 4409)		(*N* = 3598)	
	HR (95% CI)	*P*-value	HR (95% CI)	*P*-value
**Year of transplant**		.003		.020
1993 to <2005	Ref		Ref	
2005 to 2010	0.85 (0.74,0.98)		1.12 (0.92,1.37)	
> 2010 to 2019	0.78 (0.68,0.90)		0.86 (0.69,1.07)	
ATG		<.001		.296
No	Ref		Ref	
Yes	0.64 (0.55,0.74)		0.89 (0.69,1.13)	
HLA mismatch		.029		.036
5/6, 6/6	Ref		Ref	
≥ 4/6	1.14 (1.01,1.28)		1.20 (1.00,1.44)	
Age, years		.278		.041
< 5	Ref		Ref	
≥ 5 to < 12	1.02 (0.89,1.17)		1.31 (1.06,1.62)	
≥ 12	0.88 (0.72,1.08)		1.25 (0.91,1.73)	
CMV		.879		<.001
Negative	Ref		Ref	
Positive	1.01 (0.90,1.12)		0.76 (0.64,0.89)	
Diagnosis		.153		<.001
Non-malignant	Ref		Ref	
Malignant	1.11 (0.96,1.27)		0.65 (0.54,0.80)	
Pre-cryo TNCC, × 10e7/kg		.532		.539
< 2.5	Ref		Ref	
2.5-5	1.21 (0.83,1.75)		1.36 (0.76,2.45)	
>5	1.20 (0.83,1.75)		1.36 (0.74,2.47)	
Conditioning		.011		.417
MAC	Ref		Ref	
RIC	0.81 (0.68,0.96)		0.91 (0.73,1.14)	
TBI		.141		.544
No	Ref		Ref	
Yes	1.11 (0.97,1.28)		1.07 (0.86,1.33)	

Models for acute and chronic GvHD are based on multiply imputed covariate data (20 datasets) and are weighted by the inverse probability of observed acute (or chronic) GvHD outcome. See the Methods section for complete details.

Abbreviations: GvHD, graft versus host disease; HR, hazard ratio; CI, confidence interval.

### Key Observations in Disease Subgroups

#### Notable Outcomes in Patients with Leukemia

The probability of DFS at 5-years in leukemic patients improved over time. DFS increased from 34.9% in P1 (95% CI: 31.4, 38.4) to 50.2% in P3 (95% CI: 45.8, 54.4) (*P* < .0001). This was mirrored by a decrease in TRM at 1 year which was 36.4% (95% CI: 33.2, 39.6) in P1 and 18.9% (95% CI:16.3, 21.6) in P3 (*P* < .0001) (Fig. 3). GvHD and Relapse-Free Survival increased from 21.9% to 34% over the 3 time periods (Supplementary Fig. S4). There was also an increase in patients transplanted in earlier stages of the disease (*P* = .001). In multivariable analysis, TRM decreased over time (HR = 0.64, 95% CI: 0.52, 0.79 and HR = 0.51, 95% CI: 0.40, 0.65 for P2 and P3 vs. P1 respectively; *P* < .001) and with higher CD (HR = 0.65, 95% CI: 0.46, 0.94 and HR = 0.62, 95% CI: 0.42, 0.91 for 2.5-5 and >5 vs. <2.5 × 10e^7^/kg; *P* = .022). HLA mismatch (HR = 1.26, 95% CI: 1.05, 1.51 for ≤4/6 vs. 5/6 and 6/6; *P* = .011) and positive CMV serology (HR = 1.66, 95% CI: 1.39, 1.98; *P* < 0.001) were associated with increased risk of TRM. CR status (HR = 0.77, 95% CI: 0.61, 0.97 and HR = 0.79, 95% CI: 0.64, 0.98 for CR1 and CR2 vs. CR ≥ 3 or not in CR; *P* = .055), age (HR = 1.12, 95% CI: 0.89, 1.40 and HR = 1.36, 95% CI: 1.03, 1.81 for 5-12 and ≥12 vs. < 5 years old; *P* = .074), and ATG (HR = 0.8, 95% CI: 0.61, 1.03; *P* = .902) had borderline associations with TRM. Conditioning (RIC vs. MAC: HR = 1.21, 95% CI: 0.86, 1.71; *P* = .264) and TBI (HR = 0.98, 95% CI: 0.81, 1.19; *P* = .842) were unrelated to TRM in the multivariable model. The incidence of relapse, acute, and chronic GvHD did not change over time or with leukemic subtype (Supplementary Table S4).

#### Notable Outcomes in Patients with Inborn Errors of Metabolism (IEM)

Multivariable analysis of death in patients with IEM showed reduced risk over time (HR = 0.71, 95% CI: 0.52, 0.98 and HR = 0.67, 95% CI: 0.49, 0.93; *P* = .03). Increased risk of death was observed with more HLA mismatches (HR = 1.38, 95% CI: 1.03, 1.85; *P* = .022) and positive pretransplant CMV serology (HR = 1.43, 95% CI: 1.07, 1.91; *P* = .005). ATG (HR = 1.46, 95% CI: 0.77, 2.78; *P* = .23), age (HR = 0.80, 95% CI: 0.53, 1.22 and HR = 0.62, 95% CI: 0.21, 1.86 for 5-12 and ≥12 vs. < 5 years; *P* = .475), cell dose (HR = 1.17, 95% CI: 0.27, 5.07 and HR = 1.09, 95% CI: 0.26, 4.6 for 2.5-5 and >5 vs. < 2.5 × 10e7/kg; *P* = .833), and RIC vs. MAC conditioning (HR = 1.15, 95% CI: 0.68, 1.93; *P* = .625) were unrelated to death in multivariable analysis.

The incidence of neutrophil engraftment at day 60 over time ranged between 82.6% and 78.5% (*P* = .737). In multivariable analysis, positive CMV serology (HR = 0.74, 95% CI: 0.61, 0.89; *P* < .001) was negatively associated with neutrophil engraftment. There was modest evidence that higher CDs increased the probability of neutrophil engraftment (HR = 0.55, 95% CI: 0.26, 1.17 and HR = 0.79, 95% CI: 0.37, 1.70 for 2.5-5 and >5 vs. < 2.5 × 10e7/kg; *P* = .013) but caution is warranted in this interpretation due to small sample sizes. ATG use (HR = 1.26, 95% CI: 0.91, 1.76; *P* = .11), HLA mismatch (HR = 0.90, 95% CI: 0.74, 1.10; *P* = .303) and RIC vs. MAC conditioning (HR = 0.80, 95% CI: 0.58, 1.10; *P* = .156) were unrelated to neutrophil engraftment.

In multivariable analysis of cGvHD in IEM, there was an increase in risk of cGVHD in P2 vs. P1 (HR = 1.41, 95% CI: 0.97, 2.05) following by a decrease in the later period (P3 vs. P1: HR = 0.71, 95% CI: 0.46, 1.08) (*P* < .001). Other factors potentially associated with cCVHD were ATG (yes vs. no: HR = 4.36, 95% CI: 1.00, 19.10; *P* = .03) and conditioning regimen (RIC vs. MAC: HR = 0.52, 95% CI: 0.23, 1.16; *P* = .104) but these adjusted estimates were imprecise due to small sample sizes. Pre-cryo CD (HR = 2.74, 95% CI: 0.36, 20.60 and HR = 1.94, 95% CI: 0.26, 14.52 for 2.5-5 and >5 vs. <2.5 × 10e7/kg; *P* = .274), age (HR = 1.17, 95% CI: 0.78, 1.77 and HR = 1.35, 95% CI: 0.53, 3.42 for 5-12 and ≥12 vs. < 5 years old; *P* = .632), and HLA mismatch (HR = 0.85, 95% CI: 0.58, 1.25 for <4/6 vs. 5/6 and 6/6; *P* = .435) were unrelated to cGVHD in the multivariable model.

#### Notable Outcomes in Patients with ID

Multivariable analysis of death in patients with ID showed a higher risk with more HLA mismatches and older age. CI of neutrophil engraftment ranged between 79.2% and 83.7% at day 60 and was not influenced by year of transplant (*P* = .378). The CI of aGvHD decreased from 33% (95% CI: 23.7, 42.6) in P1 to 18.3% (95% CI: 13.9, 23.1) in P3 (*P* = .0048) and was impacted by year of transplant and use of ATG in multivariable analysis. The CI of cGvHD fell from 20% (12, 29.4) to 11.5% (7.2, 16.9) (*P* = .1816). In multivariable analysis there was a trend for a lower risk of cGvHD in younger age, use of ATG, closer HLA matching, and more recent time of transplant.

#### Other Key Observations

We investigated the relative contributions of HLA matching and CD in this large patient cohort. Over time, both administered CD and degree of HLA match increased. In addition, in these pediatric patients, CDs exceeded established minimal thresholds. We extracted the relative role of these 2 parameters from the multivariable analyses for patients with malignant and patients with non-malignant conditions for all-cause mortality, TRM, relapse (in patients with malignancies), neutrophil engraftment, and acute and chronic GvHD (Supplementary Fig. S5). Overall, closer HLA matching was more likely than CD to favorably influence outcomes in this group of pediatric patients receiving an adequate CD from a single CBU.

ATG was administered in the majority of transplants over time (92% of patients transplanted in P1, 77.9% in P2, and 86.4% in P3). We saw evidence that suggested the impact of ATG on TRM and all-cause mortality depended on calendar period. Specifically, ATG was associated with a trend towards reduced risk of TRM but with an increasingly larger effect over calendar time (HR = 0.6, HR = 0.35, and HR = 0.31 in P1, P2, and P3; *P* = .066). However, ATG use was associated with an increased risk of all-cause mortality in P1 that appeared to diminish in P2 and P3 (HR = 1.45, HR = 1.07, and HR = 0.99 in P1, P2, and P3; *P* = .064) (Supplementary Fig. S6).

The role of TBI in patients with leukemias in preventing relapse, increasing DFS, and contributing to TRM was also investigated. While there was no change in the incidence of relapse over time, the incidence of relapse in patients with ALL was lower when TBI was administered as part of the conditioning regimen (Supplementary Table S4).

## Discussion

We report results of CBT in a large cohort of children receiving a single unit, unrelated CBT over the past 3 decades. Several important observations are highlighted. First, OS improved over time from 41.8% before 2005 to 60% after 2010 (*P* < .0001). This is likely to be attributed to use of more closely matched and higher CD containing CB grafts. Patient related factors, including selection and treatment in earlier stages of disease, type of conditioning, selected use of post-transplant maintenance therapy, and more effective prevention and treatment modalities GvHD and infection prevention and newer and more effective treatment modalities further improved outcomes. Despite higher CDs and improved HLA matching there is still a relationship between the two in that HLA matching influenced all the outcomes including survival, TRM, engraftment, and GvHD whereas CD was important for survival and engraftment. All in all, in this population of children receiving adequate to higher doses of cells from a single CBU, closer HLA matching was more significant in favorably influencing outcomes compared to CD.

Identifying a CBU with an adequate CD, above the TNC threshold of 2.5 × 10^7^/kg, for children is not a problem due to their lower body weight. Overall, the pre-cryopreservation CB CD increased from 6.6 to 8.57 × 10^7^/kg over time. In multivariable analysis for engraftment, we found that a higher CD was associated with higher rates of engraftment compared to an inferior CD (*P* < .001) while increasing the CD above the median of TNC 6.9 × 10^7^/kg did not further improve engraftment. Thus, once this CD is reached, HLA matching should be prioritized^l,2,18,19^ Also, increasing CD beyond the median did not correlate with lower TRM or further improve OS. 

In earlier periods, CB unit selection was based on HLA-A and -B antigen typing and DR-B1 allelic typing. The number of patients with HLA-A, -B, -C, and DRB1 allele typing increased overtime representing 50% of the patients after 2010. Using low resolution typing, the number of patients with 6/6 and 5/6 HLA matching increased significantly over time to 74% after 2010 (*P* = .0001). The same trend was observed when considering patients who had high resolution typing with 79.6% of patients receiving a 5/8–8/8 matched CBU. DRB1 matching was prioritized over class I matching with 81.4% patients receiving a DRB1 matched CBU, 52% HLA-A matched, 44% HLA-B matched, and 38.4% HLA-C matched.

The complex interaction between CD and HLA parity and their impact on CBT outcomes was studied by several groups that demonstrated that increasing TNC dose resulted in higher engraftment rates and lower TRM.^20-22^ In 2007, Eapen and colleagues reported, in a pediatric cohort of 785 patients with leukemia who received unrelated bone marrow transplant or CBT, a better 5-year LFS after transplants with a 0/6 low resolution HLA mismatched CB compared to bone marrow, but no difference in engraftment of CB with 1-2/6 mismatches and bone marrow transplantation.^21^ The following year Kurtzberg *et al.* in a prospective study including 191 unrelated CBT in children with hematologic malignancies, showed improved OS with units having <2/6 mismatches, and higher TNC doses.^20^ Eapen and colleagues (2014) studied a cohort of 1568 patients (mainly children) and reported higher TRM with increasing HLA high resolution mismatches (9% for 0/8; 26% for 1-2/8; and 34 % for 3/8).^22^

CBTs have the advantage of lower incidence of acute and chronic GvHD.^23,24^ However, the impact of HLA disparity remains unclear, and many reports failed to predict the occurrence of aGvHD based on low resolution HLA disparity,^2,3,25,26^ although Rubinstein et al. showed lower frequency of severe (grades III–IV) aGvHD in patients with 6/6 HLA antigen matches (*P* = .008). In a retrospective registry-based review, the incidence of grade II–IV aGvHD was 39% after single CBT, and risk of aGvHD was found to increase with the degree of allelic HLA mismatches.^22^ Acute GvHD was also reported to correlate with an increased risk of cGvHD.^27,28^ In our cohort, the incidence of aGvHD was 33.5% similar to other published results.^22^ The correlation we found in multivariable analysis between ATG administration and risk of aGvHD was expected. However, we found no difference in the incidence of aGvHD based on HLA disparity (low- or high-resolution typing) unlike other studies that showed higher risk of developing aGvHD with mismatches at high resolution typing.^20,22^ Our findings are likely different due to the preferential selection of the CBUs with relatively high TNC doses which might be able to abrogate the effect of increasing levels of HLA mismatches. Importantly, in our cohort, aGvHD did not correlate with a high incidence of cGvHD, and only 17.5% of the children developed cGvHD at 5 years. Low resolution HLA disparity was the only factor associated with the increasing risk of cGvHD.

Positive pretransplant CMV serology appears to be an important prognostic factor in this population despite improvements in detection and treatment of CMV reactivation. Patients with a positive CMV serology before transplant had a higher risk of death, lower engraftment but a lower risk of GvHD in multivariable analysis. The association of a positive CMV serology with adverse outcomes has previously been described after HSCT from alternative donors. The combination of CB donor, which is by definition CMV negative, and the patient who could be either CMV seropositive or negative presents an opportunity to further study CMV infection and immune reconstitution after transplant.

## Conclusion

In conclusion, studying a large cohort of children transplanted with a single CBU for standard indications for HSCT over the past 3 decades, we found improved engraftment and survival and decreased TRM over time. Current CBT outcomes favorably compare with other donor sources utilized in patients lacking a matched related donor.^29–31^ Public CB bank inventories have expanded and represent prequalified, high quality, “off-the-shelf” donors that are readily available for shipment and use. When selecting a CBU for a pediatric patient, prioritize HLA-matching over CD when an adequate dose is available from their best available CBU.

## Supplementary Material

szac079_suppl_Supplementary_Material

## Data Availability

The data that support the findings of this study are available from the corresponding author upon reasonable request.

## References

[1] Broxmeyer HE , Douglas GW, Hangoc G, et al. Human umbilical cord blood as a potential source of transplantable hematopoietic stem/progenitor cells. Proc Natl Acad Sci USA. 1989;86(10):3828-3832. 10.1073/pnas.86.10.3828 2566997 PMC287234

[2] Rubinstein P , Carrier C, Scaradavou A, et al. Outcomes among 562 recipients of placental-blood transplants from unrelated donors. N Engl J Med. 1998;339(22):1565-1577. 10.1056/NEJM199811263392201 9828244

[3] Gluckman E , Rocha V, Boyer-Chammard A, et al. Outcome of cord-blood transplantation from related and unrelated donors. Eurocord Transplant Group and the European Blood and Marrow Transplantation Group. N Engl J Med. 1997;337(6):373-381. 10.1056/NEJM199708073370602 9241126

[4] Kurtzberg J , Laughlin M, Graham ML, et al. Placental blood as a source of hematopoietic stem cells for transplantation into unrelated recipients. N Engl J Med. 1996;335(3):157-166. 10.1056/NEJM199607183350303 8657213

[5] Barker JN , Weisdorf DJ, DeFor TE, et al. Transplantation of 2 partially HLA-matched umbilical cord blood units to enhance engraftment in adults with hematologic malignancy. Blood. 2005;105(3):1343-1347. 10.1182/blood-2004-07-2717 15466923

[6] Peled T , Landau E, Mandel J, et al. Linear polyamine copper chelator tetraethylenepentamine augments long-term ex vivo expansion of cord blood-derived CD34+ cells and increases their engraftment potential in NOD/SCID mice. Exp Hematol. 2004;32(6):547-555. 10.1016/j.exphem.2004.03.002 15183895

[7] Milano F , Gooley T, Wood B, et al. Cord-blood transplantation in patients with minimal residual disease. N Engl J Med. 2016;375(10):944-953. 10.1056/NEJMoa1602074 27602666 PMC5513721

[8] Wagner JE Jr , Eapen M, Carter S, et al. One-unit versus two-unit cord-blood transplantation for hematologic cancers. N Engl J Med. 2014;371(18):1685-1694. 10.1056/NEJMoa1405584 25354103 PMC4257059

[9] (WMDA) WMDA. Total number of donors and cord blood units. Accessed November 11, 2021. https://statistics.wmda.info/

[10] Horwitz ME , Stiff PJ, Cutler C, et al. Omidubicel vs standard myeloablative umbilical cord blood transplantation: results of a phase 3 randomized study. Blood. 2021;138(16):1429-1440. 10.1182/blood.2021011719 34157093 PMC9710469

[11] Keating AK , Langenhorst J, Wagner JE, et al. The influence of stem cell source on transplant outcomes for pediatric patients with acute myeloid leukemia. Blood Adv. 2019;3(7):1118-1128. 10.1182/bloodadvances.2018025908 30952678 PMC6457227

[12] Mehta RS , Holtan SG, Wang T, et al. Composite GRFS and CRFS outcomes after adult alternative donor HCT. J Clin Oncol. 2020;38(18):2062-2076. 10.1200/JCO.19.00396 32364845 PMC7302955

[13] Gutman JA , Ross K, Smith C, et al. Chronic graft versus host disease burden and late transplant complications are lower following adult double cord blood versus matched unrelated donor peripheral blood transplantation. Bone Marrow Transplant. 2016;51(12):1588-1593. 10.1038/bmt.2016.186 27400068

[14] Luznik L , O’Donnell PV, Symons HJ, et al. HLA-haploidentical bone marrow transplantation for hematologic malignancies using nonmyeloablative conditioning and high-dose, posttransplantation cyclophosphamide. Biol Blood Marrow Transplant. 2008;14(6):641-650. 10.1016/j.bbmt.2008.03.005 18489989 PMC2633246

[15] Enders CK. Multiple imputation as a flexible tool for missing data handling in clinical research. Behav Res Ther. 2017;98(11):4-18. 10.1016/j.brat.2016.11.008 27890222

[16] Seaman SR , White IR. Review of inverse probability weighting for dealing with missing data. Stat Methods Med Res. 2011;22(3):278-295. 10.1177/0962280210395740 21220355

[17] Rubin DB. Muliple Imputation for Non-Response in Surveys. Wiley and Sons, New York; 1987.

[18] Barker JN , Scaradavou A, Stevens CE. Combined effect of total nucleated cell dose and HLA match on transplantation outcome in 1061 cord blood recipients with hematologic malignancies. Blood. 2010;115(9):1843-1849. 10.1182/blood-2009-07-231068 20029048 PMC5003507

[19] Gluckman E , Rocha V, Chevret S. Results of unrelated umbilical cord blood hematopoietic stem cell transplantation. Rev Clin Exp Hematol. 2001;5(2):87-99. 10.1046/j.1468-0734.2001.00034.x 11486656

[20] Kurtzberg J , Prasad VK, Carter SL, et al. Results of the Cord Blood Transplantation Study (COBLT): clinical outcomes of unrelated donor umbilical cord blood transplantation in pediatric patients with hematologic malignancies. Blood. 2008;112(10):4318-4327. 10.1182/blood-2007-06-098020 18723429 PMC2581998

[21] Eapen M , Rubinstein P, Zhang MJ, et al. Outcomes of transplantation of unrelated donor umbilical cord blood and bone marrow in children with acute leukaemia: a comparison study. Clinical Trial, Comparative Study, Multicenter Study. Research Support, N.I.H., Extramural Research Support, U.S. Gov’t, Non-P.H.S. Lancet. 2007;369(9577):1947-1954. 10.1016/S0140-6736(07)60915-5 17560447

[22] Eapen M , Klein JP, Ruggeri A, et al. Impact of allele-level HLA matching on outcomes after myeloablative single unit umbilical cord blood transplantation for hematologic malignancy. Blood. 2014;123(1):133-140. 10.1182/blood-2013-05-506253 24141369 PMC3879902

[23] Eapen M , Rocha V, Sanz G, et al. Effect of graft source on unrelated donor haemopoietic stem-cell transplantation in adults with acute leukaemia: a retrospective analysis. Lancet Oncol. 2010;11(7):653-660. 10.1016/S1470-2045(10)70127-3 20558104 PMC3163510

[24] Brunstein CG , Gutman JA, Weisdorf DJ, et al. Allogeneic hematopoietic cell transplantation for hematologic malignancy: relative risks and benefits of double umbilical cord blood. Blood. 2010;116(22):4693-4699. 10.1182/blood-2010-05-285304 20686119 PMC2996124

[25] Gluckman E , Rocha V, Arcese W, et al. Factors associated with outcomes of unrelated cord blood transplant: guidelines for donor choice. Exp Hematol. 2004;32(4):397-407. 10.1016/j.exphem.2004.01.002 15050751

[26] Locatelli F , Rocha V, Chastang C, et al. Factors associated with outcome after cord blood transplantation in children with acute leukemia. Eurocord-Cord Blood Transplant Group. Blood. 1999;93(11):3662-3671. 10339472

[27] Chen YB , Wang T, Hemmer MT, et al. GvHD after umbilical cord blood transplantation for acute leukemia: an analysis of risk factors and effect on outcomes. Bone Marrow Transplant. 2017;52(3):400-408. 10.1038/bmt.2016.265 27941764 PMC5332289

[28] Lazaryan A , Weisdorf DJ, DeFor T, et al. Risk factors for acute and chronic graft-versus-host disease after allogeneic hematopoietic cell transplantation with umbilical cord blood and matched sibling donors. Biol Blood Marrow Transplant. 2016;22(1):134-140. 10.1016/j.bbmt.2015.09.008 26365153 PMC4787268

[29] Sharma P , Purev E, Haverkos B, et al. Adult cord blood transplant results in comparable overall survival and improved GRFS vs matched related transplant. Blood Adv. 2020;4(10):2227-2235. 10.1182/bloodadvances.2020001554 32442301 PMC7252552

[30] Ponce DM , Zheng J, Gonzales AM, et al. Reduced late mortality risk contributes to similar survival after double-unit cord blood transplantation compared with related and unrelated donor hematopoietic stem cell transplantation. Biol Blood Marrow Transplant. Sep 2011;17(9):1316-1326. 10.1016/j.bbmt.2011.01.006 21232625 PMC3156939

[31] Xue E , Milano F. Are we underutilizing bone marrow and cord blood? Review of their role and potential in the era of cellular therapies. F1000Res. 2020;9:26. 10.12688/f1000research.20605.1 PMC6970216 31984133

