# Supplementary material for: Unrelated Donor Cord Blood Transplantation in Children: Lessons Learned Over 3 Decades
Source: Stem Cells Transl Med. 2023 Jan 30;12(1):26–38. doi: 10.1093/stcltm/szac079 (PMC9887081; doi:10.1093/stcltm/szac079)

**Supplemental Figure 1: Changes in HLA Matching and Cell Dose Over Time**

**Supplemental Figure 2: Overall Transplant Outcomes**

**Supplemental Figure 3: Overall Survival in Selected Disease Groups**

**Supplemental Figure 4: GvHD and Relapse-Free Survival by year of Transplant, Malignancies only**

**Supplemental Figure 5: Relative Importance of HLA Matching and Pre-Cryopreservation Cell Dose**

**Supplemental Figure 6. The Changing Impact of ATG on All-Cause and Transplant-Related Mortality for Malignancies**

**Supplemental Table 1: Causes of Death by Year of Transplant**

**Supplemental Table 2a: Causes of Transplant Related Mortality by Year of Transplant**

**Supplemental Table 2b: Results of Fine & Gray Proportional Hazards Regression Modeling of Transplant-Related Mortality, Malignancies Only**

**Supplemental Table 3: Results of Fine and Gray Proportional Hazards Regression Modeling of Chronic Graft Versus Host Disease (GvHD) After Transplant for Malignant and Non-Malignant Disease**

**Supplemental Table 4: Results of Fine & Gray Proportional Hazards Regression Modeling of Relapse**

**Figure Legends:**

Supplemental Figure 1. Changes in HLA Matching and Cell Dose over Time: (A) The proportion of transplants matched at >5/6 loci are shown in red and the proportion of transplants matched at <4/6 loci are shown in blue. The proportion of patients with 5/6 or 6/6 matched donor cord blood units (shaded in red) increased over time. (B) The cell dose delivered by the cord blood unit graft increased over time.

Supplemental Figure 2: Overall Transplant Outcomes: Overall survival at 10 years (A), Neutrophil Engraftment at 60 days(B), Acute GvHD at 100 days (C) and Chronic GvHD at 10 years(D) are shown for the entire patient cohort.

Supplemental Figure 3: Overall Survival in Selected Disease Groups: Overall survival in the entire cohort at 10 years is show by diagnosis. (A) survival in patients with Acute Leukemia, (B) survival in patients with Inborn Errors of Metabolism, (C) survival in patients with Immune Deficiencies, and (D) survival in patients with Aplastic Anemia.

Supplemental Figure 4: GvHD and Relapse-Free Survival (GRFS) by Year of Transplant, Malignancies only: GRFS increased significantly significantly between period 1 and periods 2 and 3.

Supplemental Figure 5. Relative Importance of HLA Matching and Precryopreservation Cell Dose: The impact of HLA matching and precryopreservation cell dose on key clinical outcomes including TRM, relapse, engraftment, acute and chronic GvHD indicating whether HLA and or cell dose had a statistically significant impact on each outcome. If the variable was statistically significant, it is shown in Green. Comparing HLA and Cell dose, HLA influenced more outcomes than cell dose. Of note, this analysis was conducted in a population of children who received an adequate dose of cells from their cord blood graft.

Supplemental Figure 6. The Changing Impact of ATG on All-Cause and Transplant-Related Mortality for Malignancies: All-cause mortality (left) and transplant related mortality related to ATG was examined by time period. Both risks decreased over time.

**Supplemental Table 1: Causes of Death by Year of Transplant**

|  | **1993 to < 2005 (N=721)** | **2005 to 2010 (N=757)** | **> 2010 to 2019 (N=611)** | **Total (N=2,089)** |
| --- | --- | --- | --- | --- |
| **Cause of Death** |  |  |  |  |
| Relapse/disease progression | 220 (30.5%) | 225 (29.7%) | 183 (30.0%) | 628 (30.1%) |
| Transplant related | 443 (61.4%) | 438 (57.9%) | 339 (55.5%) | 1220 (58.4%) |
| Secondary malignancy | 4 (0.6%) | 2 (0.3%) | 6 (1.0%) | 12 (0.6%) |
| Other cause(s) | 12 (1.7%) | 19 (2.5%) | 17 (2.8%) | 48 (2.3%) |
| Unknown/Not Reported | 42 (5.8%) | 73 (9.6%) | 66 (10.8%) | 181 (8.7%) |

**Supplemental Table 2a. Causes of Transplant Related Mortality by Year of Transplant**

|  | **1993 to < 2005 (N=443)** | **2005 to 2010 (N=438)** | **> 2010 to 2019 (N=339)** | **Total (N=1,220)** |
| --- | --- | --- | --- | --- |
| **Transplant-Related Mortality** |  |  |  |  |
| Missing | 4 | 5 | 7 | 16 |
| GvHD | 80 (18.2%) | 81 (18.7%) | 75 (22.6%) | 236 (19.6%) |
| idiopathic pneumonia syndrome | 28 (6.4%) | 25 (5.8%) | 9 (2.7%) | 62 (5.1%) |
| VOD | 17 (3.9%) | 19 (4.4%) | 14 (4.2%) | 50 (4.2%) |
| Hemorrhage | 24 (5.5%) | 23 (5.3%) | 19 (5.7%) | 66 (5.5%) |
| Rejection | 22 (5.0%) | 25 (5.8%) | 6 (1.8%) | 53 (4.4%) |
| Bacterial infection | 29 (6.6%) | 25 (5.8%) | 38 (11.4%) | 92 (7.6%) |
| Viral infection | 52 (11.8%) | 49 (11.3%) | 34 (10.2%) | 135 (11.2%) |
| Fungal infection | 36 (8.2%) | 18 (4.2%) | 9 (2.7%) | 63 (5.2%) |
| Parasitic infection | 5 (1.1%) | 2 (0.5%) | 3 (0.9%) | 10 (0.8%) |
| Unknown infection | 32 (7.3%) | 59 (13.6%) | 52 (15.7%) | 143 (11.9%) |
| Cardiac toxicity | 7 (1.6%) | 14 (3.2%) | 7 (2.1%) | 28 (2.3%) |
| ARDS | 25 (5.7%) | 22 (5.1%) | 10 (3.0%) | 57 (4.7%) |
| Secondary malignancy | 0 (0.0%) | 0 (0.0%) | 1 (0.3%) | 1 (0.1%) |
| Multiorgan failure | 51 (11.6%) | 38 (8.8%) | 16 (4.8%) | 105 (8.7%) |
| LPTD EBV | 14 (3.2%) | 10 (2.3%) | 2 (0.6%) | 26 (2.2%) |
| other | 15 (3.4%) | 16 (3.7%) | 35 (10.5%) | 66 (5.5%) |
| Unknown | 2 (0.5%) | 7 (1.6%) | 2 (0.6%) | 11 (0.9%) |

**Supplemental Table 2b. Results of Fine & Gray Proportional Hazards Regression Modeling of Transplant-Related Mortality, Malignancies Only (N=2,802)**

|  | **HR (95% CI)** | **P-value** |
| --- | --- | --- |
| **Year of Transplant** |  | <0.001 |
| 1993 to < 2005 | Ref |  |
| 2005 to 2010 | 0.58 (0.49,0.70) |  |
| > 2010 to 2019 | 0.47 (0.39,0.58) |  |
| **ATG** |  | 0.066 |
| No | Ref |  |
| Yes | 0.82 (0.65,1.02) |  |
| **HLA Mismatch** |  | 0.002 |
| 5/6, 6/6 | Ref |  |
| <= 4/6 | 1.28 (1.09,1.50) |  |
| **Age, Years** |  | 0.011 |
| < 5 | Ref |  |
| >= 5 to < 12 | 1.13 (0.93,1.37) |  |
| >= 12 | 1.43 (1.12,1.83) |  |
| **CMV** |  | <0.001 |
| Negative | Ref |  |
| Positive | 1.60 (1.37,1.86) |  |
| **Pre-cryo TNCC, x10e7/kg** |  | 0.018 |
| < 2.5 | Ref |  |
| 2.5 - 5 | 0.66 (0.48,0.91) |  |
| > 5 | 0.64 (0.46,0.90) |  |
| **Conditioning** |  | 0.375 |
| MAC | Ref |  |
| RIC | 1.13 (0.84,1.53) |  |
| **TBI** |  | 0.189 |
| No | Ref |  |
| Yes | 0.89 (0.76,1.06) |  |

**Supplemental Table 3: Results of Fine and Gray Proportional Hazards Regression Modeling of Chronic Graft Versus Host Disease (GvHD) After Transplant for Malignant and Non-Malignant Disease**

|  | **Malignant**  **(N=2,042)** | | | **Non-Malignant**  **(N=1,387)** | | |
| --- | --- | --- | --- | --- | --- | --- |
|  | **HR (95% CI)** | **P-value** | **HR (95% CI)** | | **P-value** |  |
| **Year of Transplant** |  | 0.614 |  | | <0.001 |  |
| 1993 to < 2005 | Ref |  | Ref | |  |  |
| 2005 to 2010 | 0.89 (0.70,1.13) |  | 1.30 (1.00,1.69) | |  |  |
| > 2010 to 2019 | 0.95 (0.73,1.23) |  | 0.75 (0.56,1.00) | |  |  |
| **ATG** |  | 0.021 |  | | 0.650 |  |
| No | Ref |  | Ref | |  |  |
| Yes | 0.73 (0.54,0.98) |  | 1.05 (0.74,1.49) | |  |  |
| **HLA Mismatch** |  | <0.001 |  | | 0.698 |  |
| 5/6, 6/6 | Ref |  | Ref | |  |  |
| <= 4/6 | 1.49 (1.20,1.84) |  | 0.96 (0.74,1.24) | |  |  |
| **Age, Years** |  | 0.685 |  | | 0.021 |  |
| < 5 | Ref |  | Ref | |  |  |
| >= 5 to < 12 | 1.11 (0.86,1.42) |  | 1.40 (1.06,1.86) | |  |  |
| >= 12 | 1.04 (0.75,1.44) |  | 1.72 (0.98,2.99) | |  |  |
| **CMV** |  | <0.001 |  | | 0.144 |  |
| Negative | Ref |  | Ref | |  |  |
| Positive | 0.70 (0.58,0.86) |  | 0.85 (0.69,1.06) | |  |  |
| **Pre-cryo TNCC, x10e7/kg** |  | 0.222 |  | | 0.423 |  |
| < 2.5 | Ref |  | Ref | |  |  |
| 2.5 - 5 | 1.52 (0.79,2.90) |  | 1.73 (0.70,4.30) | |  |  |
| > 5 | 1.64 (0.85,3.18) |  | 1.55 (0.63,3.80) | |  |  |
| **Conditioning** |  | 0.914 |  | | 0.331 |  |
| MAC | Ref |  | Ref | |  |  |
| RIC | 0.99 (0.66,1.49) |  | 0.89 (0.71,1.13) | |  |  |
| **TBI** |  | 0.082 | -- | | -- |  |
| No | Ref |  | -- | | -- |  |
| Yes | 1.22 (0.97,1.54) |  | -- | | -- |  |

Models for malignant and non-malignant disease are based on multiply imputed covariate data (20 datasets) and are weighted by the inverse probability of observed chronic GvHD outcome. See the Methods section for complete details.

**Supplemental Table 4: Results of Fine & Gray Proportional Hazards Regression Modeling of Relapse**

|  | **Acute Leukemia***  **(N=2,243)** | | **AML**  **(N=837)** | | **ALL**  **(N=1,373)** | |
| --- | --- | --- | --- | --- | --- | --- |
|  | **HR (95% CI)** | **P-value** | **HR (95% CI)** | **P-value** | **HR (95% CI)** | **P-value** |
| **Year of Transplant** |  | 0.815 |  | 0.920 |  | 0.820 |
| 1993 to < 2005 | Ref |  | Ref |  | Ref |  |
| 2005 to 2010 | 1.03 (0.82,1.28) |  | 1.04 (0.71,1.53) |  | 1.06 (0.81,1.39) |  |
| > 2010 to 2019 | 1.08 (0.85,1.36) |  | 1.10 (0.73,1.65) |  | 1.10 (0.82,1.47) |  |
| **ATG** |  | 0.478 |  | 0.085 |  | 0.678 |
| No | Ref |  | Ref |  | Ref |  |
| Yes | 1.09 (0.83,1.43) |  | 1.49 (0.87,2.55) |  | 0.93 (0.67,1.29) |  |
| **HLA Mismatch** |  | 0.098 |  | 0.243 |  | 0.148 |
| 5/6, 6/6 | Ref |  | Ref |  | Ref |  |
| <= 4/6 | 0.85 (0.70,1.03) |  | 0.82 (0.59,1.15) |  | 0.84 (0.65,1.08) |  |
| **Age, Years** |  | 0.002 |  | 0.002 |  | 0.182 |
| < 5 | Ref |  | Ref |  | Ref |  |
| >= 5 to < 12 | 0.70 (0.57,0.88) |  | 0.60 (0.41,0.88) |  | 0.78 (0.59,1.02) |  |
| >= 12 | 0.66 (0.49,0.88) |  | 0.38 (0.22,0.67) |  | 0.89 (0.62,1.27) |  |
| **CMV** |  | 0.095 |  | 0.848 |  | 0.050 |
| Negative | Ref |  | Ref |  | Ref |  |
| Positive | 0.87 (0.73,1.03) |  | 0.99 (0.73,1.32) |  | 0.80 (0.64,1.00) |  |
| **Pre-cryo TNCC, x10e7/kg** |  | 0.446 |  | 0.154 |  | 0.617 |
| < 2.5 | Ref |  | Ref |  | Ref |  |
| 2.5 - 5 | 1.25 (0.75,2.08) |  | 1.21 (0.49,3.02) |  | 1.27 (0.69,2.33) |  |
| > 5 | 1.12 (0.67,1.90) |  | 0.81 (0.31,2.15) |  | 1.32 (0.71,2.43) |  |
| **Conditioning** |  | 0.364 |  | 0.944 |  | 0.159 |
| MAC | Ref |  | Ref |  | Ref |  |
| RIC | 0.82 (0.54,1.25) |  | 1.02 (0.57,1.84) |  | 0.65 (0.35,1.20) |  |
| **TBI** |  | 0.259 |  | 0.706 |  | 0.022 |
| No | Ref |  | Ref |  | Ref |  |
| Yes | 0.89 (0.73,1.09) |  | 1.08 (0.74,1.59) |  | 0.75 (0.58,0.96) |  |
| **CR Status** |  | <0.001 |  | <0.001 |  | <0.001 |
| CR >=3 or Not in CR | Ref |  | Ref |  | Ref |  |
| CR 1 | 0.34 (0.26,0.43) |  | 0.28 (0.19,0.41) |  | 0.35 (0.26,0.49) |  |
| CR 2 | 0.50 (0.41,0.62) |  | 0.45 (0.31,0.64) |  | 0.51 (0.38,0.67) |  |

*The number of AML + ALL doesn’t equal the total number of acute leukemia because there are other subtypes of leukemia not shown in the table.

**Supplemental Figure 1: Changes in HLA Matching and Pre-Cryopreservation Cell Dose**

**
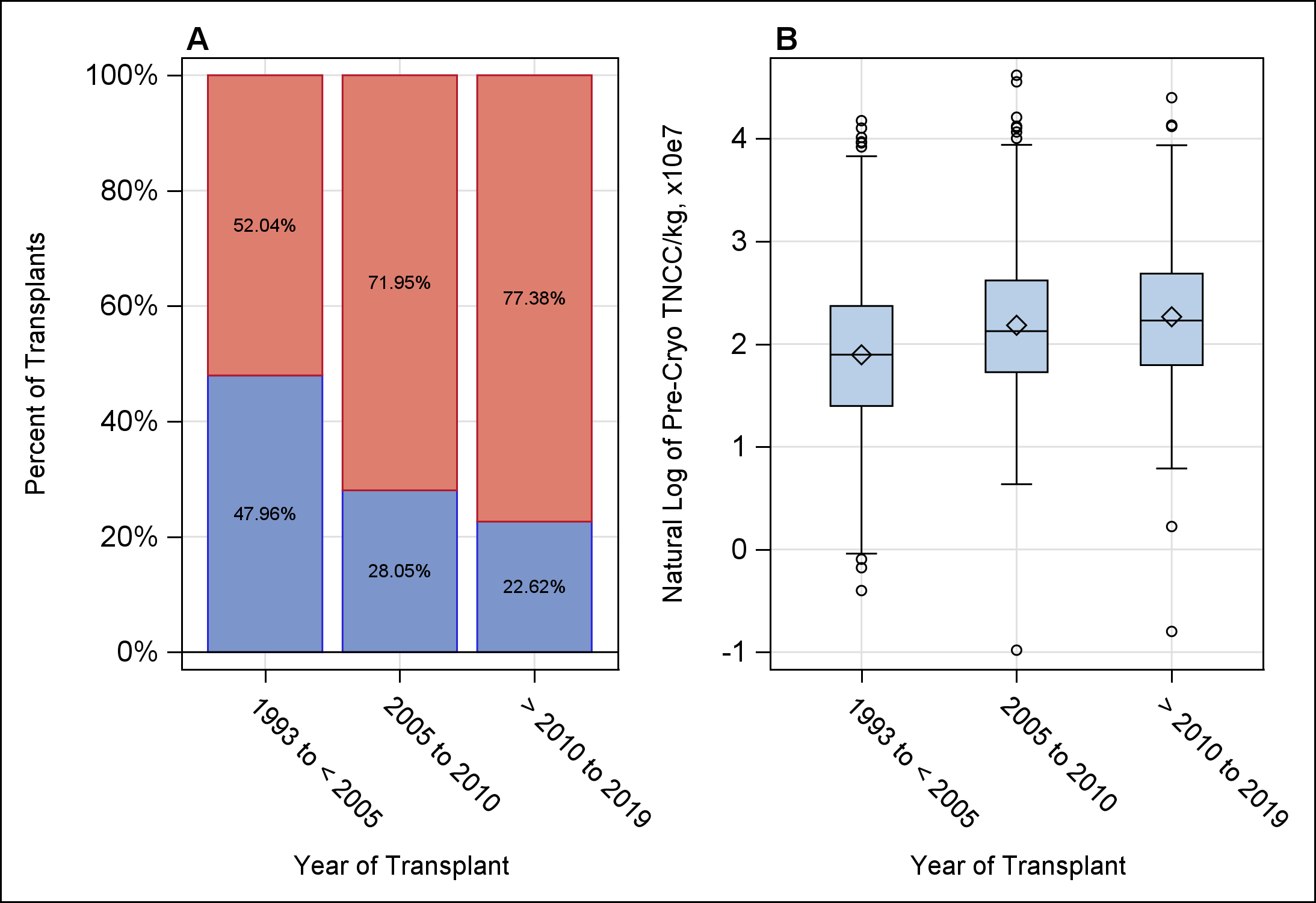
**

**Supplemental Figure 2: Overall Transplant Outcomes**

**
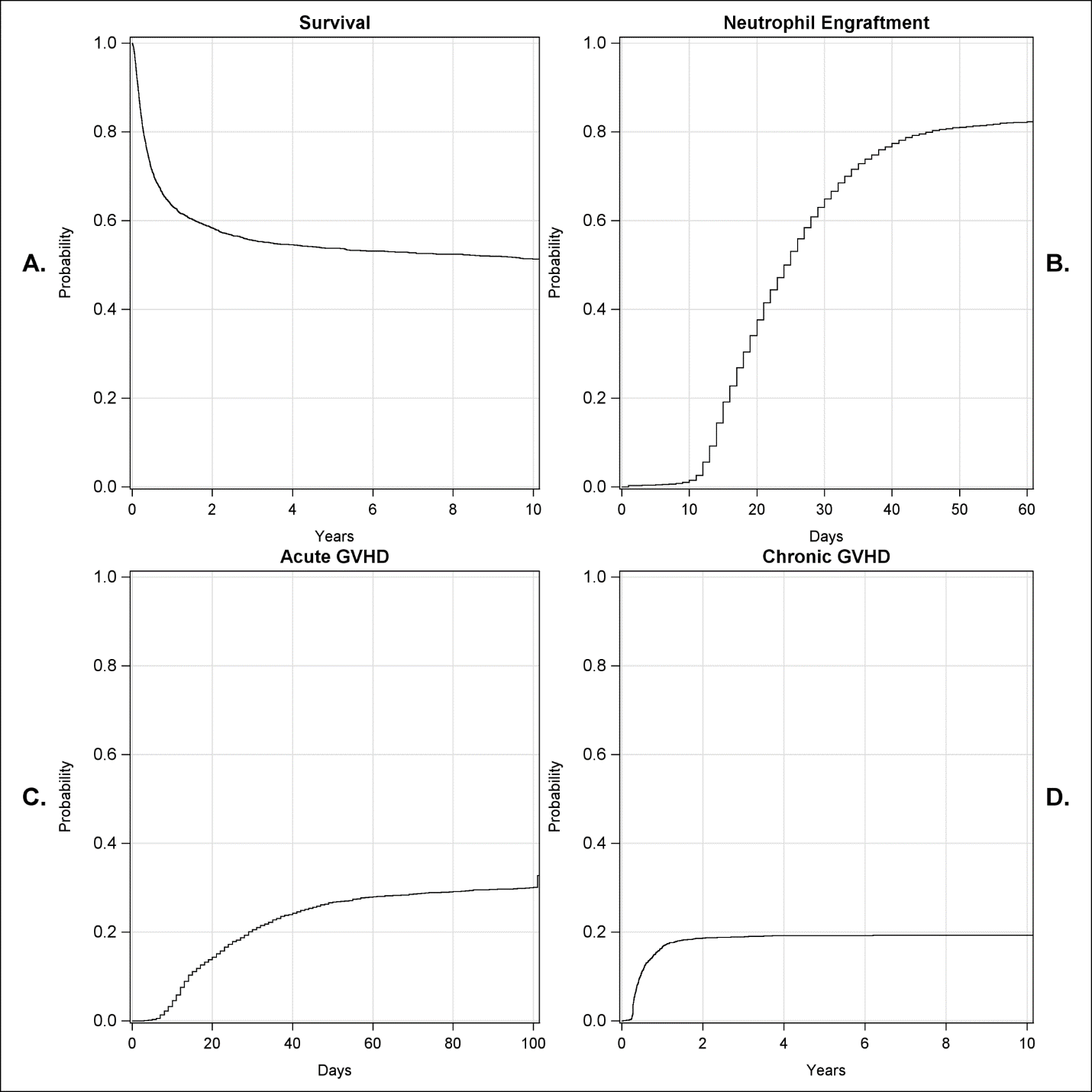
**

**Supplemental Figure 3: Overall Survival in Selected Disease Groups**

**
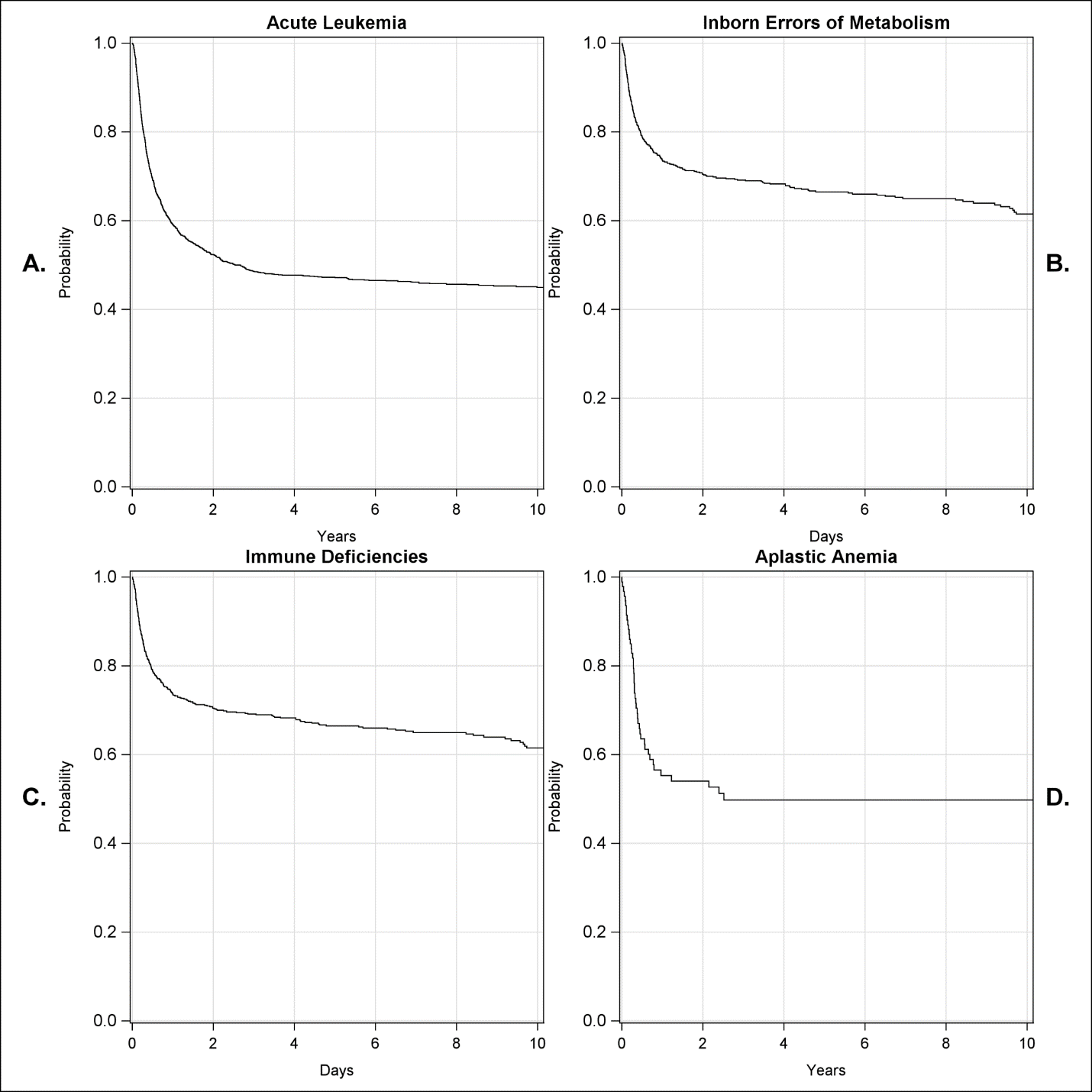
**

**Supplemental Figure 4: GvHD and Relapse-Free Survival By Year of Transplant, Malignancies Only**


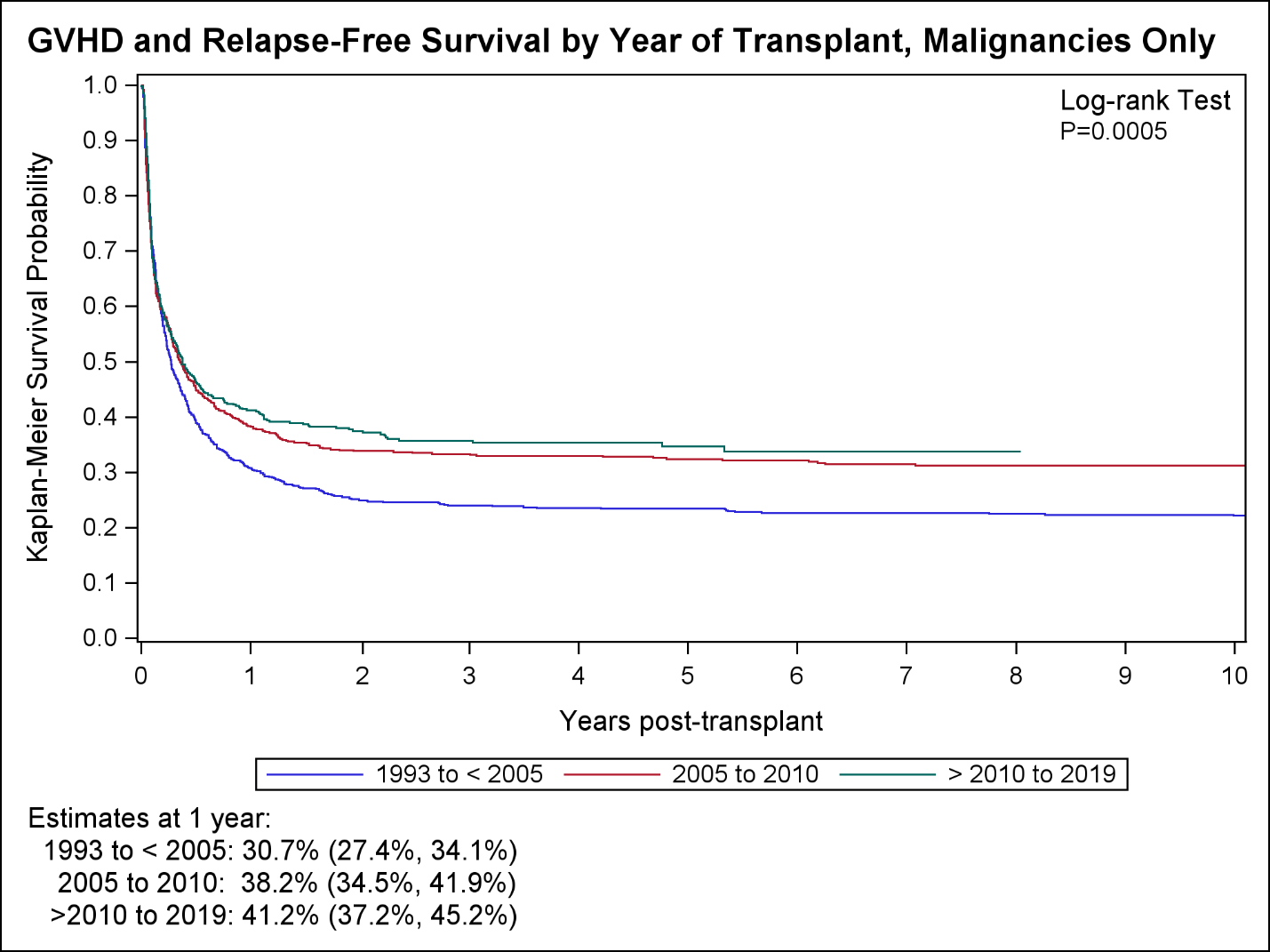


**Supplemental Figure 5: Relative Importance of HLA Matching and Pre-Cryopreservation Cell Dose**


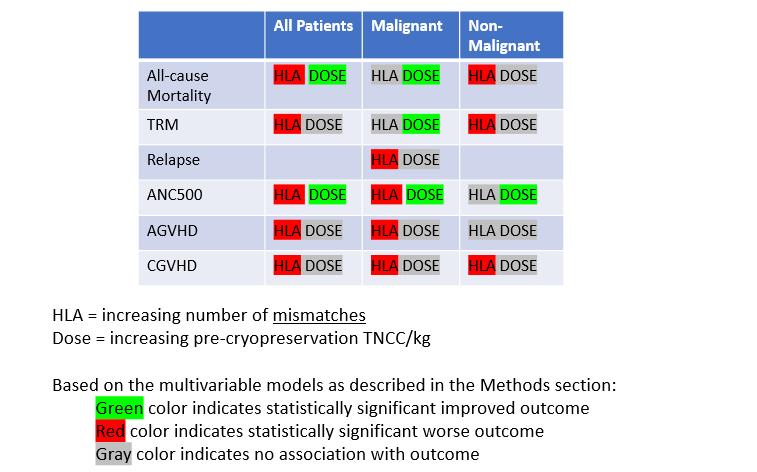


**Supplemental Figure 6. The Changing Impact of ATG on All-Cause and Transplant-Related Mortality for Malignancies**


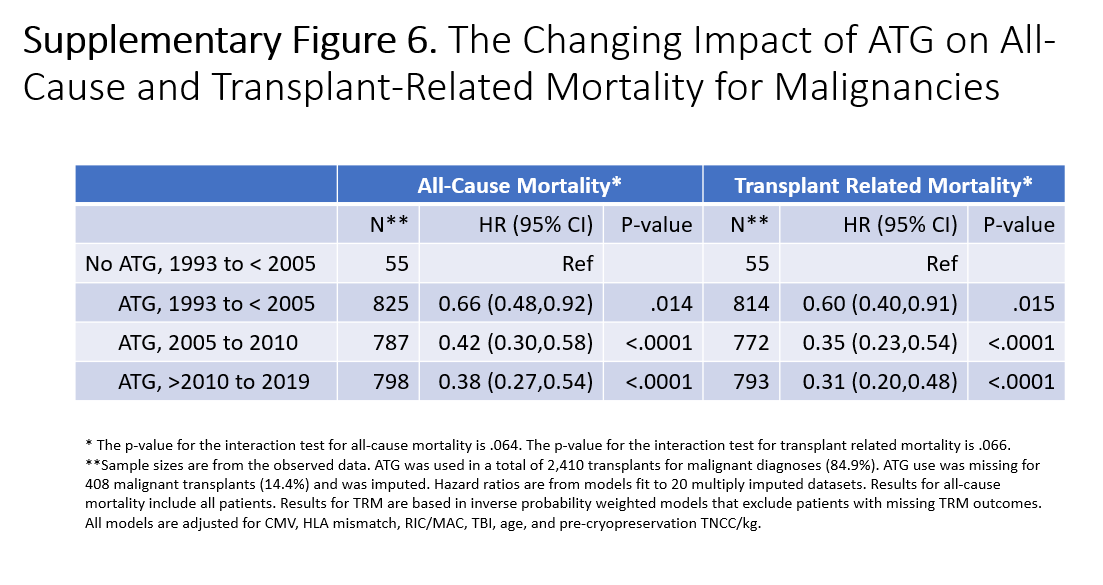

Supplement: szac079_suppl_Supplementary_Material [file szac079_suppl_supplementary_material.docx]
